# Sarcomatoid carcinoma of the jejunum presenting as obscure gastrointestinal bleeding in a patient with a history of gliosarcoma

**DOI:** 10.1093/gastro/gou007

**Published:** 2014-02-27

**Authors:** Nidia Alfonso Puentes, Carmen Jimenez-Alfaro Larrazabal, Maria Isabel García Higuera

**Affiliations:** ^1^Clinical Neurophysiology Department, Hospital Universitario de Burgos, Spain; ^2^Department of Internal Medicine, Hospital Universitario de Burgos, Spain; ^3^Department of Pathology, Hospital Universitario de Burgos, Spain

**Keywords:** obscure gastrointestinal bleeding, small bowel tumors, sarcomatoid carcinoma

## Abstract

Small bowel malignant tumors are rare and sarcomatoid carcinomas have rarely been reported at this site. We report a 56-year-old woman, with history of an excised gliosarcoma, who presented with recurrent obscure gastrointestinal bleeding. She underwent endoscopy and colonoscopy, which failed to identify the cause of the bleeding. The abdominal computed tomography scan located a tumor in the small bowel. Pathology revealed a jejunal sarcomatoid carcinoma. She developed tumor recurrence and multiple liver metastases shortly after surgery. Immunohistochemistry is required for accurate diagnosis. Sarcomatoid carcinoma is a rare cause of obscure gastrointestinal bleeding, which is associated with a poor prognosis.

## INTRODUCTION

The American Gastroenterological Association defines obscure gastrointestinal bleeding (OGIB) as bleeding of unknown origin that persists or recurs without an obvious etiology after a normal endoscopy (colonoscopy and upper endoscopy). It can be categorized into overt gastrointestinal (GI) bleeding with recurrent melena or hematochezia and occult GI bleeding with iron deficiency anemia and/or positive result on fecal blood test [1, 2].

OGIB accounts for approximately 5% of patients with GI bleeding. In approximately 75% of these patients, the source is in the small bowel, which is difficult to assess with conventional endoscopic and radiological procedures [1, 3]. Evaluation of the patient with OGIB is dependent on the extent of the bleeding and the age of the patient. A second-look esophago-gastro-duodenoscopy and colonoscopy should be considered in all patients with occult or recurrent overt bleeding, owing to the high rate of missed lesions. Capsule endoscopy (CE) is currently recommended as the third test of choice, after a normal bidirectional endoscopy. Double balloon enteroscopy (DBE) is indicated if CE detects a lesion requiring biopsy or endoscopic intervention, or in patients in whom suspicion of small bowel bleeding is high despite a negative initial CE. In those patients in whom CE is contra-indicated, and in patients in whom a tumor is suspected, computed tomography enterography (CTE) may be the preferred initial test for small bowel evaluation [2–4].

The introduction of CE and DBE and the recent improvements in computed tomography (CT) and magnetic resonance imaging (MRI) techniques have revolutionized the approach to patients with OGIB, allowing the visualization of the entire GI tract, particularly the small bowel—until now considered as the ‘dark continent’ [3]. We describe a rare case of OGIB, the origin of which was found in a jejunal sarcomatoid carcinoma (SCA), in a patient with a history of gliosarcoma.

## CASE REPORT

A 56-year-old woman with a past history of a right temporal gliosarcoma (excised in April 2011 followed by postoperative radiotherapy, with no evidence of recurrence) was admitted on two occasions in May 2012, with asthenia and two previous episodes of coffee ground vomiting. An initial upper endoscopy showed no signs of active bleeding. One month later, she was re-admitted due to persistent anemia and a 3–4 kg weight loss. On physical examination, she was pale. Rectal examination was normal. Laboratory results showed: hematocrit, 18.3%; hemoglobin, 5.7 g/dL; serum iron, 10 μg/dL (37–45); erythrocyte sedimentation rate, 131 mm/h; c-reactive protein, 143 mg/L; AST, 103 U/L; ALT, 87 U/L; gamma-GT, 189 U/L; alkaline phosphatase, 573 U/L; total bilirubin, 0.6 mg/dL. The rest of the laboratory tests, including tumor markers, were normal.

**Figure 1.**
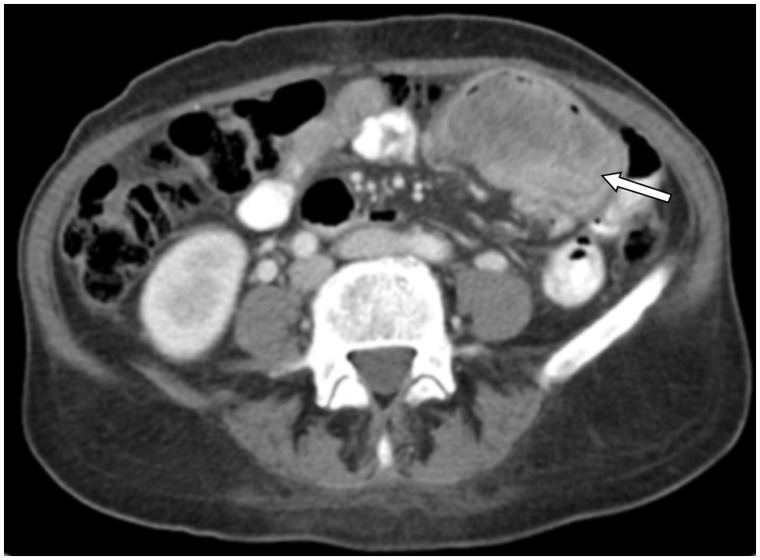
Abdominal CT scan showing a mass arising from the small bowel.

A colonoscopy was performed but showed no abnormal findings. An abdominal ultrasound showed a solid C-shaped mass, 9 cm in diameter, at the level of the pelvis, probably arising from the small bowel. A subsequent abdominal/pelvic CT scan (Figure 1) showed a solid, heterogeneous mass, 6.7 × 4.4 cm, arising from the small bowel. No infiltration of adjacent structures or the presence of enlarged lymph nodes was observed. Chest CT scan detected no distant spread. She required multiple blood transfusions before surgery. A wide resection of the small intestine was performed.

**Figure 2.**
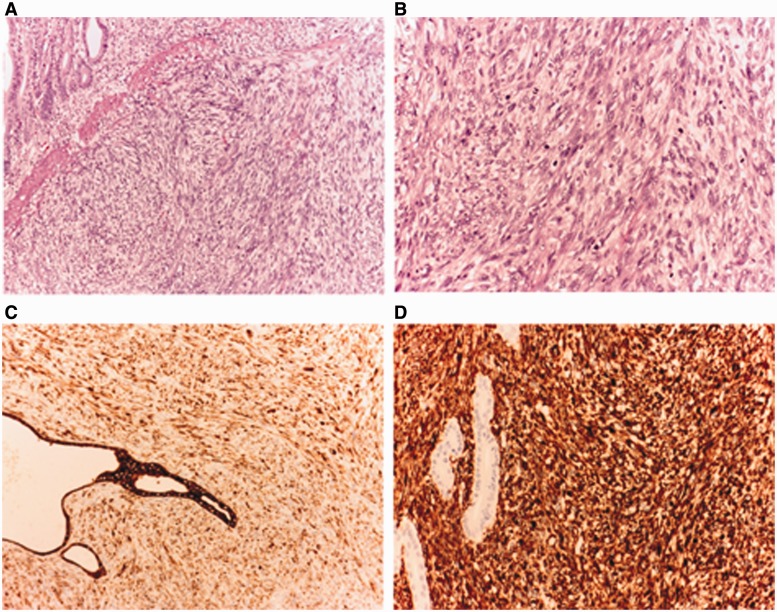
(A) Tumor of sarcomatoid appearance in the small bowel wall. (HE × 100). (B) Fascicular proliferation with moderate atypia and increased mitotic activity. (HE × 200). (C) Tumor cells are positive for cytokeratin (AE1/AE3 and CAM 5.2) with a positive control in the preserved intestinal mucosa. (CAM 5.2 × 200). (D) Tumor cells are also positive for stromal marker, which is negative in the preserved intestinal mucosa. (Vimentin × 200).

Macroscopic examination of the specimen revealed a 10 × 6.5 cm small bowel mass that was extensively ulcerated and had areas of necrosis. Microscopically, the tumor showed sheets of pleomorphic spindle cells arranged in fascicles with vesicular nuclei, small nucleoli, and eosinophilic cytoplasm. There was no evidence of epithelial differentiation. Mitotic figures averaged 35–40 per 10 high-power fields. The surgical margins were free of involvement. The tumor extended through the wall of the jejunum into the surrounding tissue and extensively invaded the serosa. Eleven lymph nodes were dissected from the mesentery, three of them showing presence of tumor deposits (TNM stage pT3 pN1 M0). Immunohistochemistry revealed strong positive staining for vimentin and cytokeratin (AE1/AE3 and CAM 5.2). Epithelial membrane antigen (EMA) was focal positive. Other markers were negative (CD 117 (c-Kit), S-100 protein, DOG 1, CD 34, smooth muscle actin (SMA), caldesmon and desmin. The diagnosis of jejunal SCA was made (Figure 2).

Adjuvant chemotherapy was planned following surgery. Two months later the patient developed multiple liver metastases. After four months of regular follow-up, left-sided hemiparesis recurred and MRI revealed tumor recurrence in the right temporal lobe. A body CT scan showed a recurrent small bowel mass, with abdominal disease progression. She died six months after diagnosis.

## DISCUSSION

The etiology of OGIB is strictly age-dependent, with angiodysplasia and drug-induced small bowel injury being most common in patients more than 40 years of age. Among patients younger than 40 years, small bowel tumors, Crohn’s disease, Meckel diverticulum, and polyposis syndromes are the most likely causes [1, 3]. Angiodysplasia represents the most common cause of OGIB (50–70%). It commonly occurs in the elderly, but has also been shown to be common in patients with chronic kidney disease [3].

Small bowel tumors are the second most common cause of OGIB, accounting for 5–10% of cases. Several different types of tumor can arise within the small bowel, both malignant (adenocarcinoma, carcinoid, lymphoma and sarcoma), and benign (adenoma, leiomyoma and lipoma) [3, 5, 6]. Small bowel cancers are uncommon and difficult to diagnose because of the non-specific symptoms. Gastrointestinal Stromal Tumors (GISTs) are often associated with rapid bleeding, whereas adenocarcinomas, carcinoids, and lymphomas cause a more gradual and chronic blood loss. Melanoma-, lung-, breast- and renal-cell carcinomas may spread to the small bowel [3]. Malignant melanoma shows an unusual predilection to metastasize to the small bowel [3, 5, 7].

Non-steroidal anti-inflammatory drugs are the most frequent cause of drug-induced small bowel injury and the damage mainly occurs in the ileocecal region. The injury, even when not severe, can cause persistent bleeding and iron deficiency anemia. Generally, there is 2–10 mL of daily blood loss. Frank, acute GI bleeding is relatively rare and is caused by ulcers and erosions [8]. Other less common causes of OGIB include Crohn’s disease, Meckel’s diverticulum, vasculitis, Dieulafoy’s lesion, hemobilia, hemosuccus pancreaticus, and aorto-enteric fistula in patients who underwent a surgical abdominal aneurysm repair [1, 3].

Our patient had a gliosarcoma, which is a rare primary tumor of the brain, classified by the World Health Organization as a high-grade glioma and a variant of glioblastoma multiforme, consisting of both gliomatous and sarcomatous components. Multiple reports in the literature describe the greater tendency of gliosarcoma to metastasize to extracranial (commonly lungs, liver, and lymph nodes) and intra-axial sites (cervical spine). There are reported cases of metastases to skin, chest wall, pleura, spleen, kidneys, bone marrow, adrenal and thyroid gland, pericardium, myocardium, diaphragm, stomach and pancreas. Gliosarcoma has a poor prognosis, due mainly to high recurrence rates [9]. In our case, local recurrence occurred 18 months after craniotomy. In the published literature, we did not find any reference to gliosarcoma that had metastasized to the small bowel.

SCA is a rare variant of small intestinal carcinoma, which displays both carcinomatous and sarcomatous features [10, 11]. Gastrointestinal SCA has been described in the gall bladder, stomach, esophagus and colon, but is only rarely reported in the small bowel, with less than 30 cases reported to date in the literature in English [10]. Within the small bowel, they primarily occur in the ileum, followed by the jejunum, with the duodenum being the site in ones case [11, 12]. Histologically, SCA may appear with a biphasic or monophasic pattern. A mixture of epithelial-looking and mesenchymal-like cells characterizes the typical biphasic pattern; monophasic tumors show a predominance of mesenchymal component, with minimal or absent epithelial components, as was seen in our case [10–12].

Because monophasic SCA can be confused with other sarcomas due to the absence of carcinomatous features, a wide-range panel of immunohistochemical biomarkers should be performed for the differential diagnosis [10]. Leiomyosarcoma presents with positivity for SMA and desmin, while these markers are negative in SCA. Negativity for DOG1, C-kit and CD34 ruled out a GIST. Lack of S-100 protein ruled out a schwannoma. SCA shows positivity for cytokeratin and vimentin, but negativity for SMA, desmin, CD 117, CD 34, and S-100 protein, as we found in our patient. In this case, the carcinomatous nature of the tumor was evident only after the immunohistochemistry test.

Small bowel carcinomas have a poor prognosis, but that for SCA is far worse, because they are highly aggressive tumors and most patients present with advanced disease [10–12]. Our patient developed liver metastases and local recurrence shortly after diagnosis, which confirmed the aggressive nature of the tumor. Surgery remains the mainstay of treatment; neither radiotherapy nor chemotherapy affects the survival rate [10–12].

After a search in PubMed using the words, ‘sarcomatoid carcinoma’ and ‘gliosarcoma’, we have not, to date, found any previously published case associating both tumors, so we consider that this association was coincidental in our patient. We cannot exclude the possibility that the patient had an unrecognized cancer predisposition syndrome (e.g. Li Fraumeni syndrome, etc.) given the fact that she developed two rare types of tumor, and her father died of lung cancer at the age of 58.

In summary, both gliosarcoma and SCA are very rare tumors with high tendency to metastasize because of the sarcomatous component, resulting in a high mortality and recurrence rates. SCA of the small intestine must be kept in mind in the management of obscure GI bleeding.

**Conflict of interest:** none declared.
